# The burden of interstitial lung disease and pulmonary sarcoidosis lung cancer among adolescents and young adults from 1990 to 2021 and its projections: a comparative study between China and other G20 countries

**DOI:** 10.3389/fmed.2025.1680118

**Published:** 2025-12-10

**Authors:** Long Chen, Jiuhe Wang, Jinglian Qu

**Affiliations:** 1 Guizhou University of Traditional Chinese Medicine, Guiyang, China; 2 Inner Mongolia Autonomous Region Hospital of Traditional Chinese Medicine, Hohhot, China

**Keywords:** interstitial lung disease, lung cancer, disease burden, temporal trends, decomposition analysis, forecasting, age-standardized rates

## Abstract

**Purpose:**

Interstitial lung disease and pulmonary sarcoidosis (ILD and PS) and lung cancer represent significant global health threats. Limited research has examined the disease burden and temporal trends of ILD and PS lung cancer among adolescents and young adults (aged 15–39 years). This study aimed to assess the burden of ILD and PS lung cancer in China, analyze contributing factors, compare this burden with other G20 nations, and forecast future disease trends in China.

**Methods:**

For ILD and PS lung cancer in China, we established incidence, prevalence, mortality, and disability-adjusted life years (DALYs) by executing data from the Global Burden of Diseases, Injuries, and Risk Factors Study (GBD) 2021. Analyses that were comparative to other G20 nations were carried out. Estimated annual percentage changes (EAPCs) were used to assess temporal trends in age-standardized rates (ASRs). Decomposition analysis quantified drivers of burden changes. Furthermore, autoregressive integrated moving average (ARIMA) models projected incidence, mortality, and DALY rates for ILD and PS lung cancer in China from 2021 to 2050.

**Results:**

Between 1990 and 2021, China experienced a steady decline in the age-standardized prevalence rate (ASPR), incidence rate (ASIR), death rate (ASDR), and disability-adjusted life years (DALY) rate for ILD and PS. The estimated annual percentage change (EAPC) values were −2.5 (95% CI: –2.9 to −2.1) for ASPR, −2.1 (95% CI: –2.5 to–1.8) for ASIR, –0.55 (95% CI: –0.71 to −0.39) for ASDR, and –1.2 (95% CI: −1.3 to −1.2) for the age-standardized DALY rate. According to 2021 data, among G20 nations, China ranked 19th in ASPR and ASIR for ILD and PS, while it ranked 20th in both ASDR and DALY rate. In contrast, the burden of lung cancer remained consistently high. With the exception of ASPR, which ranked second, all other burden indicators for lung cancer were the highest among G20 countries, with corresponding EAPCs of 0.22 (95% CI: 0.02 to 0.41) for ASPR, −0.52 (95% CI: –0.71 to −0.34) for ASIR, −0.98 (95% CI: –1.18 to–0.78) for ASDR, and −1.01 (95% CI:–1.19 to −0.83) for the age-standardized DALY rate. Decomposition analysis further revealed that shifts in epidemiological patterns were the key factor curbing the rise in absolute numbers of prevalence, incidence, deaths, and DALYs for ILD and PS in China, whereas population growth served as the primary driver increasing the prevalence and incidence of lung cancer. Model-based projections indicate that over the next 29 years, the ASPR, ASIR, ASDR, and age-standardized DALY rate for both ILD and PS lung cancer in China are expected to continue declining.

**Conclusion:**

ILD and PS lung cancer impose substantial epidemiological burdens across G20 countries, including China. This pressing reality necessitates coordinated multinational efforts to establish collaborative research frameworks, integrate healthcare resources, optimize early screening protocols, and jointly develop evidence-based prevention and control strategies to address this critical public health challenge.

## 1 Introduction

Interstitial Lung Disease (ILD and PS) encompasses over 200 types of diffuse lung diseases. The core pathophysiological feature of this disease group is the involvement of the lung interstitium and alveolar cavities, which subsequently leads to the loss of the alveolar-capillary functional unit. The typical clinical features include progressive dyspnea, restrictive ventilatory dysfunction accompanied by a decrease in carbon monoxide diffusion capacity, hypoxemia, and diffuse bilateral lung lesions on imaging. As the disease progresses to the terminal stage, patients often die due to irreversible respiratory failure caused by damage to the lung structure. Epidemiological studies have revealed significant age, gender, race, and regional differences in the incidence of ILD and PS. Among the classification of ILD and PS, idiopathic pulmonary fibrosis (IPF) is one of the common types in clinical practice. IPF mainly affects male individuals over 60 years old. Its annual incidence rate is 0.9–9.3 per 100,000 people in Europe and North America, while it is 3.5–13.0 per 100,000 people in Asia and South America. In contrast, patients with idiopathic nonspecific interstitial pneumonia (iNSIP) and connective tissue disease-related interstitial lung disease (CTD-ILD and PS) are more than 50% middle-aged or elderly women aged 40–60. Overall, the estimated prevalence of ILD and PS ranges from 6.3 to 76.0 per 100,000 people (1). The incidence and prevalence rates of this disease have been increasing year by year among the population (2). This disease progresses rapidly and has a poor prognosis. The median survival period after diagnosis is only 2 to 3 years (3). Currently, the clinical treatment of ILD and PS mainly involves anti-fibrotic drugs (4). Immunomodulatory therapy (5) and mainly involving lung transplantation (6), although it can to some extent improve the quality of life of patients, it still has limitations in delaying the progression of the disease and reducing the mortality rate.

Lung cancer, as the leading cause of cancer-related deaths worldwide, exhibits highly aggressive biological characteristics (7). The global cancer statistics for 2020 show that there were 1.8 million deaths due to lung cancer, accounting for approximately 20% of all cancer-related deaths (8). Since the 1990s, lung cancer has remained the leading cause of cancer-related mortality in China, accounting for more than one-third of global lung cancer deaths. Moreover, approximately 50% of the world’s newly recorded lung cancer deaths over the past three decades have occurred within the Chinese population (9). Lung cancer is classified histopathologically into two primary types: small cell lung cancer (SCLC) and non-small cell lung cancer (NSCLC). Population-based registries reveal substantial geographic variations in incidence; between 2009 and 2018, lung cancer rates declined in the United States (10), remained stable in Switzerland for both SCLC and NSCLC (11), while exhibiting a steady increase within the Chinese population (12). Despite the clinical implementation of various novel therapeutic strategies, including targeted inhibition of mutated tyrosine kinase pathways (13), immunotherapy (14), and novel biologic agents (15), suboptimal overall efficacy has been observed.

More critically, patients with advanced-stage lung cancer exhibit significantly poorer survival outcomes compared to those with other common malignancies. Furthermore, epidemiological research on lung cancer in China is predominantly derived from region-specific data and often characterized by relatively short study periods, which hinders comprehensive trend analysis (16).

Accumulating evidence indicates that the risk of lung cancer in patients with ILD and PS ranges from approximately 10 to 20%, representing a 3.5-to 7.3-fold increase compared with the general population (17). Therefore, ILD and PS constitutes not only a significant cause of respiratory disability but also an independent risk factor for lung cancer development. Elucidating the disease burden and epidemiological trends of both ILD and PS lung cancer has urgent clinical significance for optimizing early screening strategies in high-risk populations and developing interdisciplinary diagnostic and therapeutic pathways.

The Group of Twenty (G20), as one of the world’s largest and most influential forums for international economic cooperation, represents approximately two-thirds of the global population (18). In terms of the research background, the distribution and evolution of disease burden are profoundly influenced by the social and economic environment as well as the healthcare conditions (19). Previous studies have identified significant geographical disparities and a socioeconomic gradient in the disease burden of ILD and PS lung cancer. However, a systematic assessment comparing China with other G20 members regarding the levels and long-term trends of ILD and PS lung cancer burden remains lacking. A comprehensive understanding of the current status and future trajectory of this burden is crucial for enhancing disease prevention and control efforts and aiding health policymakers in optimally allocating limited healthcare resources. Building on this, leveraging data from the Global Burden of Disease Study 2021 (GBD 2021), this study aims to quantitatively analyze trends in the incidence, prevalence, mortality, and disability-adjusted life years (DALYs) for ILD and PS lung cancer in China from 1990 to 2021. This analysis includes a cross-comparison with other G20 nations over the same period, while also projecting the burden trends for both diseases in China through 2050.

## 2 Methods

The following address deals with access to the information table and representation of effects: https://vizhub.healthdata.org/gbd-results. According to ICD-10, ILD and PS is a group of long-term respiratory conditions that affect oxygen exchange and pulmonary function and are characterized by inflammation and/or scarring. J84 being particularly relevant for “Other interstitial pulmonary diseases.” Non-small cell lung cancer (NSCLC) and small cell lung cancer (SCLC) are the two main histological subtypes of lung cancer, which is defined as malignancies that originate from the bronchial mucosa or pulmonary glands (ICD-10: C34). Publicly available aggregated data from the Global Burden of Diseases, Injuries, and Risk Factors Study (GBD2021) was used in this secondary analysis. This extensive dataset, which includes 87 behavioral, environmental, occupational, and metabolic risk factors, methodically measures burden metrics for 369 diseases and injuries across 204 countries/territories (1990–2021). Global health repositories, vital registration systems, sample registrations, censuses, household surveys, surveillance sites, and published literature (systematic reviews, cohort studies, cross-sectional surveys, and case reports) were among the data sources. For ILD and PS cancer in China, we retrieved age-standardized incidence, prevalence, deaths, and DALYs (1990–2021). Argentina, Australia, Brazil, Canada, France, Germany, India, Indonesia, Italy, Japan, Mexico, South Korea, Russia, Saudi Arabia, South Africa, Turkey, the United Kingdom, the United States, and the European Union (as a whole) were the other G20 countries against which these metrics were compared. DALYs, which are the total of years of life lost (YLL) and years lived with disability (YLD), are a comprehensive measure of health loss from premature mortality and disability according to GBD methodology. One DALY is equivalent to 1 year of lost healthy life.

## 3 Statistical analysis

Burden metrics-including incidence counts, prevalence, mortality, and disability-adjusted life years (DALYs) - were reported as absolute numbers, percentages, and rates. All estimates incorporated 95% uncertainty intervals (UI). Within the GBD framework, each metric (e.g., incident cases, age-standardized rates) underwent 1,000 computational iterations. These iterations accounted for sampling variability across data inputs, transformations, and model specifications. The range between the 25th and 975th sorted values from these iterations is represented by the 95% UI. The direct method was used to generate age-standardized rates (ASRs) for incidence, prevalence, mortality, and DALYs, standardizing to the World Health Organization’s (WHO) 2000–2025 world standard population. From 1990 to 2021, we calculated expected annual percentage changes (EAPCs) in ASRs to measure temporal trends. Fitting the natural logarithm of rates to calendar year using linear regression yielded the EAPC, a validated metric for describing rate trends: ln(rate) = *α* + *β* × (calendar year) + *ε*, where EAPC = 100 × (exp(β) – 1). Due of the regression model, the 95% CI for EAPC was established. Trend interpretations were based on the following standards: When the EAPC and its lower 95% CI limit both surpassed zero, a rising trend was identified. When the EAPC and its upper 95% CI limit were both negative, a declining trend was verified.

The decomposition analysis aims to dissect the constituent elements of composite health indicators (such as incidence rate, prevalence rate, mortality rate, and age-standardized DALYs), in order to clarify the contribution of each component to the overall result. Specifically, this method can quantitatively attribute the changes in disease burden to different influencing factors, such as changes in population age structure, population size fluctuations, and changes in epidemiological patterns. To investigate the evolution of the burden of ILD and PS cancer, this study adopts the decomposition analysis framework proposed by Das Gupta and combines it with the improved method proposed by Li et al. (20). This combined approach decomposes the overall changes in disease burden into three group-level determinants: population aging, population growth, and epidemiological changes (21). The core principle can be summarized as follows. The number of disease burden indicators (X) at each location is obtained using the following formula:


$$X_{\mathrm{ay},\mathrm{py},\mathrm{ey}}=\sum_{\left(i=1\right)}^{17}(a_{i,y}*P_{y}*e_{i,y})$$

Here, a_i,y_ stand for the percentage of the population in age category I in the given year y; p_y_ for the total population in year y; and e_i,y_ for the ASR in age category I in year y. These indicators of disease burden are based on the age structure, population, and ASR factors for a particular year y. The impact of a factor change while other factors stayed constant characterized each element’s contribution to the shift in disease burden between 1990 and 2021. The overall change in the disease burden indicator should be precisely equal to the sum of the effects of each driving element.

The ARIMA model, known as the Autoregressive Integrated Moving Average model, represents a widely used approach for time series forecasting. In the ARIMA (p,d,q) notation, AR stands for “autoregressive” with p indicating the number of autoregressive terms. MA denotes “moving average” where q represents the number of lagged forecast errors in the model. The parameter d indicates the degree of differencing applied to render the time series stationary. For comprehensive details regarding the ARIMA model, please refer to the Supplementary materials (22). The model was constructed through the following sequential steps: stationarity testing, model identification, parameter estimation, model diagnostics, and forecasting. The Augmented Dickey-Fuller (ADF) test was employed to assess stationarity of the time series. When non-stationarity was detected, logarithmic transformation was applied to achieve stationarity. Appropriate model parameters (p and q) were determined by analyzing the autocorrelation function (ACF) and partial autocorrelation function (PACF). The optimal ARIMA (p,d,q) model was selected using both Akaike’s Information Criterion (AIC) and Bayesian Information Criterion (BIC). Model adequacy was verified through Ljung-Box Q-test of residuals, along with examination of residual ACF and PACF plots. The finalized model was subsequently utilized for forecasting. All statistical analyses and graphical representations were performed using the R software package, with statistical significance defined as *p* < 0.05.

## 4 Results

### 4.1 Disease burden of ILD and PS cancer in China

In China, prevalent ILD and PS cases decreased from 39,420 (95% UI: 23,452–58,943) in 1990 to 24,795 (95% UI: 15,423–36,889) in 2021. Globally, prevalent cases increased from 211,818 (95% UI: 135,334–301,745) in 1990 to 265,217 (95% UI: 176,703–369,506) in 2021. China’s ASPR declined from 8.0 (95% UI: 4.8–11.8) per 100,000 in 1990 to 4.6 (95% UI: 2.8–7.0) in 2021. This was consistently lower than the global average (10.7 [95% UI: 6.9–15.2] in 1990; 8.9 [95% UI: 5.9–12.4] in 2021). Among G20 nations, China ranked 16th for ASPR in 1990 and 19th in 2021, surpassed only by Indonesia (Table 1) (The first place equals the highest value. The result section is as follows). Incident ILD and PS cases in China decreased from 5,171 (95% UI: 2,532–8,810) in 1990 to 3,280 (95% UI: 1,611–5,599) in 2021. China’s ASIR ranking among G20 nations improved from 18th in 1990 to 19th in 2021 (Table 2). ILD and PS deaths in China decreased from 162 (95% UI: 110–267) in 1990 to 132 (95% UI: 81–198) in 2021. China’s ASDR ranking rose from 19th among G20 nations in 1990 to 20th in 2021 (Table 3). Globally, ILD and PS related DALYs were 149,440 thousand (95% UI: 113,475–194,398) in 1990 and 236,946 thousand (95% UI: 190,514–282,730) in 2021. In 1990, the United States had the highest age-standardized DALY rate (18.5 [95% UI: 16.9–20.7] per 100,000), while China ranked 18th (2.8 [95% UI: 2.0–4.1]). By 2021, Saudi Arabia had the highest rate (27.2 [95% UI: 15.5–44.6]), and China had the lowest rate among G20 countries (2.0 [95% UI: 1.4–2.9]) (Table 4).

**Table 1:** Prevalence of ILD and PS in G20 countries and global average, and temporal trends from 1990 to 2021.

Location	Cases		1990–2021 change (%)	Age-standardized prevalence rate, per 100000 (95% UI)		1990–2021 EAPC (95% CI)
	1990	2021		1990	2021	
China	39,420 (23,452, 58,943)	24,795 (15,423, 36,889)	−37.10%	8.0 (4.8, 11.8)	4.6 (2.8, 7.0)	−2.5 (−2.9, −2.1)
Global	211,818 (135,334, 301,745)	265,217 (176,703, 369,506)	25.21%	10.7 (6.9, 15.2)	8.9 (5.9, 12.4)	−0.6 (−0.6, −0.5)
Indonesia	2,110 (1,105, 3,506)	4,130 (2,335, 6,537)	95.73%	3.1 (1.7, 5.1)	3.6 (2.0, 5.7)	0.5 (0.5, 0.6)
Russian Federation	9,180 (5,968, 12,854)	6,125 (4,085, 8,464)	−33.28%	14.5 (9.4, 20.4)	11.1 (7.3, 15.5)	−0.9 (−1.0, −0.8)
Japan	13,332 (8,702, 18,623)	9,320 (6,609, 12,446)	−30.09%	30.0 (19.6, 42.0)	26.6 (18.8, 35.6)	−0.2 (−0.4, −0.1)
Republic of Korea	5,606 (3,877, 7,625)	5,547 (3,870, 7,552)	−1.05%	27.9 (19.4, 37.9)	31.1 (21.6, 42.5)	0.4 (0.3, 0.5)
Australia	409 (241, 616)	707 (430, 1,036)	72.86%	5.9 (3.5, 9.0)	7.5 (4.5, 11.0)	0.8 (0.7, 0.8)
France	1,693 (1,012, 2,487)	1,636 (1,002, 2,393)	−3.37%	7.5 (4.5, 11.0)	8.0 (4.9, 11.7)	0.3 (0.2, 0.3)
Germany	2,354 (1,408, 3,478)	2,220 (1,357, 3,232)	−5.69%	7.5 (4.5, 11.2)	7.8 (4.8, 11.5)	1.0 (0.4, 1.7)
Italy	3,644 (2,276, 5,249)	2,601 (1772, 3,557)	−28.62%	17.6 (11.0, 25.3)	15.2 (10.3, 20.8)	−0.6 (−0.8, −0.4)
United Kingdom	3,987 (2,561, 5,673)	2,925 (1959, 4,104)	−26.64%	19.3 (12.5, 27.5)	12.5 (8.4, 17.6)	−1.0 (−1.3, −0.8)
Argentina	1,319 (849, 1896)	2,263 (1,488, 3,183)	71.57%	11.4 (7.4, 16.4)	12.9 (8.5, 18.1)	0.4 (0.4, 0.4)
Canada	2,809 (1894, 3,880)	3,132 (2,140, 4,256)	11.50%	23.5 (15.8, 32.6)	24.4 (16.6, 33.2)	0.2 (0.1, 0.2)
United States of America	34,453 (23,111, 47,307)	30,441 (21,324, 40,673)	−11.64%	31.9 (21.4, 43.8)	26.6 (18.6, 35.6)	−0.6 (−0.8, −0.3)
Mexico	2,975 (1842, 4,346)	4,183 (2,825, 5,850)	40.61%	10.3 (6.4, 14.8)	8.3 (5.6, 11.6)	−0.6 (−1.0, −0.2)
Brazil	6,068 (3,859, 8,697)	4,171 (2,704, 6,124)	−31.26%	10.8 (6.9, 15.4)	4.7 (3.0, 6.9)	−2.6 (−3.2, −2.1)
Saudi Arabia	609 (375, 883)	3,578 (2,414, 4,937)	487.52%	10.5 (6.6, 15.1)	16.2 (10.9, 22.5)	1.6 (1.5, 1.7)
Turkey	2092 (1,302,3,040)	3,917 (2,526, 5,573)	87.24%	10.1 (6.3, 14.5)	12.0 (7.8, 17.1)	1.0 (0.7, 1.3)
India	27,408 (16,563, 40,103)	61,982 (39,861, 87,974)	126.15%	9.1 (5.6, 13.3)	10.7 (6.9, 15.1)	0.8 (0.6, 1.0)
South Africa	1,670 (1,069, 2,386)	2,667 (1732, 3,767)	59.70%	12.5 (8.1, 17.7)	10.6 (6.8, 15.0)	−0.7 (−0.8, −0.5)
European Union	19,578 (12,754, 27,583)	17,250 (11,687, 23,716)	−11.89%	12.2 (8.0, 17.2)	12.0 (8.1, 16.6)	0.1 (−0.1, 0.4)

**Table 2:** Incidence of ILD and PS in G20 countries and global average, and temporal trends from 1990 to 2021.

Location	Cases		1990–2021 change (%)	Age-standardized incidence rate, per 100000 (95% UI)		1990–2021 EAPC (95% CI)
	1990	2021		1990	2021	
China	5,171 (2,532, 8,810)	3,280 (1,611, 5,599)	−36.57%	1.0 (0.5, 1.7)	0.6 (0.3, 1.1)	−2.1 (−2.5, −1.8)
Global	27,744 (14,935, 44,541)	34,320 (19,101, 53,991)	23.70%	1.4 (0.7, 2.2)	1.2 (0.6, 1.8)	−0.5 (−0.6, −0.4)
Indonesia	307 (126, 572)	562 (252, 1,008)	83.06%	0.4 (0.2, 0.8)	0.5 (0.2, 0.9)	0.5 (0.2, 0.7)
Russian Federation	982 (535, 1,562)	574 (309, 922)	−41.55%	1.6 (0.9, 2.6)	1.2 (0.6, 1.9)	−1.1 (−1.2, −1.0)
Japan	1,524 (837, 2,416)	963 (558, 1,478)	−36.81%	3.5 (1.9, 5.4)	2.9 (1.7, 4.3)	−0.6 (−0.6, −0.5)
Republic of Korea	630 (373, 965)	566 (329, 873)	−10.16%	3.0 (1.8, 4.7)	3.3 (1.9, 5.1)	0.4 (0.3, 0.6)
Australia	50 (22, 86)	83 (41, 140)	66%	0.7 (0.3, 1.3)	0.9 (0.5, 1.5)	0.7 (0.6, 0.9)
France	229 (117, 378)	215 (109, 353)	−6.11%	1.0 (0.5, 1.7)	1.1 (0.5, 1.7)	0.3 (0.2, 0.4)
Germany	325 (167, 546)	289 (148, 481)	−11.08%	1.0 (0.5, 1.8)	1.0 (0.5, 1.8)	0.9 (0.3, 1.6)
Italy	450 (239, 726)	300 (168, 467)	−33.33%	2.1 (1.1, 3.5)	1.8 (1.0, 2.8)	−0.6 (−0.9, −0.3)
United Kingdom	555 (311, 859)	403 (224, 624)	−27.39%	2.7 (1.5, 4.1)	1.7 (1.0, 2.7)	−1.0 (−1.3, −0.8)
Argentina	177 (94, 284)	297 (164, 467)	67.80%	1.5 (0.8, 2.4)	1.7 (0.9, 2.7)	0.4 (0.3, 0.5)
Canada	340 (190, 526)	374 (213, 570)	10%	2.9 (1.6, 4.5)	3.0 (1.7, 4.6)	0.1 (0.0, 0.2)
United States of America	4,320 (2,461, 6,638)	3,751 (2,216, 5,641)	−13.17%	4.1 (2.3, 6.3)	3.3 (1.9, 5.0)	−0.7 (−0.9, −0.6)
Mexico	435 (225, 706)	603 (336, 940)	38.62%	1.4 (0.7, 2.2)	1.2 (0.7, 1.9)	-0.6 (−0.9, −0.2)
Brazil	791 (423, 1,264)	505 (251, 855)	−36.16%	1.3 (0.7, 2.1)	0.6 (0.3, 1.0)	−2.6 (−3.2, −2.0)
Saudi Arabia	85 (44, 138)	476 (271, 723)	460%	1.4 (0.7, 2.3)	2.2 (1.3, 3.4)	1.7 (1.6, 1.8)
Turkey	272 (140, 446)	467 (250, 752)	71.69%	1.2 (0.6, 2.0)	1.5 (0.8, 2.3)	1.1 (0.8, 1.4)
India	4,159 (2,171, 6,730)	8,917 (4,936, 13,889)	114.40%	1.3 (0.7, 2.1)	1.5 (0.8, 2.4)	0.6 (0.4, 0.9)
South Africa	236 (127, 373)	342 (187, 539)	44.92%	1.7 (0.9, 2.6)	1.4 (0.7, 2.2)	−0.8 (−0.9, −0.6)
European Union	2,393 (1,310, 3,812)	1981 (1,116, 3,108)	−17.22%	1.5 (0.8, 2.4)	1.4 (0.8, 2.2)	0.0 (−0.3, 0.3)

**Table 3:** Deaths of ILD and PS in G20 countries and global average, and temporal trends from 1990 to 2021.

Location	Cases		1990–2021 change (%)	Age-standardized deaths rate, per 100000 (95% UI)		1990–2021 EAPC (95% CI)
	1990	2021		1990	2021	
China	162 (110, 267)	132 (81, 198)	−18.52%	0.03 (0.02, 0.05)	0.03 (0.02, 0.04)	−0.55 (−0.71, −0.39)
Global	2,121 (1,571, 2,821)	3,519 (2,769, 4,230)	65.91%	0.10 (0.08, 0.14)	0.12 (0.09, 0.14)	0.42 (0.36, 0.48)
Indonesia	24 (5, 79)	49 (12, 140)	104.17%	0.03 (0.01, 0.11)	0.04 (0.01, 0.12)	1.16 (1.03, 1.29)
Russian Federation	56 (44, 67)	22 (20, 25)	−60.71%	0.09 (0.07, 0.11)	0.04 (0.04, 0.04)	−3.91 (−4.77, −3.04)
Japan	43 (41, 45)	36 (35, 38)	−16.28%	0.10 (0.09, 0.10)	0.11 (0.10, 0.11)	0.10 (−0.22, 0.42)
Republic of Korea	10 (5, 19)	9 (5, 14)	−10%	0.05 (0.02, 0.09)	0.05 (0.03, 0.08)	0.52 (0.32, 0.71)
Australia	2 (2, 3)	12 (10, 14)	500%	0.03 (0.03, 0.04)	0.13 (0.10, 0.15)	4.51 (3.46, 5.57)
France	12 (10, 14)	21 (17, 25)	75%	0.05 (0.04, 0.06)	0.10 (0.09, 0.12)	2.75 (2.32, 3.19)
Germany	17 (14, 22)	21 (18, 25)	23.53%	0.06 (0.04, 0.07)	0.08 (0.07, 0.09)	2.23 (1.82, 2.64)
Italy	5 (4, 5)	20 (19, 21)	300%	0.02 (0.02, 0.02)	0.12 (0.12, 0.13)	6.33 (4.87, 7.82)
United Kingdom	13 (13, 14)	45 (43, 47)	246.15%	0.06 (0.06, 0.07)	0.20 (0.19, 0.20)	5.26 (4.37, 6.15)
Argentina	28 (23, 34)	36 (30, 43)	28.57%	0.24 (0.20, 0.29)	0.20 (0.17, 0.25)	−0.10 (−0.44, 0.24)
Canada	12 (10, 15)	26 (22, 31)	116.67%	0.11 (0.09, 0.13)	0.21 (0.18, 0.25)	2.39 (2.06, 2.71)
United States of America	277 (267, 288)	245 (230, 261)	−11.55%	0.26 (0.25, 0.27)	0.22 (0.20, 0.23)	−0.61 (−0.72, −0.50)
Mexico	55 (52, 58)	177 (158, 198)	221.82%	0.18 (0.17, 0.19)	0.35 (0.31, 0.39)	2.25 (1.96, 2.55)
Brazil	100 (92, 109)	183 (171, 196)	83%	0.17 (0.16, 0.19)	0.21 (0.19, 0.22)	−0.10 (−0.49, 0.29)
Saudi Arabia	17 (8, 33)	90 (49, 152)	429.41%	0.28 (0.13, 0.53)	0.43 (0.23, 0.73)	2.06 (1.75, 2.38)
Turkey	20 (8, 38)	28 (15, 47)	40%	0.09 (0.04, 0.17)	0.09 (0.05, 0.15)	0.57 (0.24, 0.90)
India	517 (218,929)	1,003 (611, 1,444)	94.00%	0.17 (0.07, 0.30)	0.17 (0.10, 0.25)	0.00 (−0.26, 0.25)
South Africa	32 (16, 46)	33 (23, 52)	3.13%	0.24 (0.12, 0.34)	0.13 (0.09, 0.21)	−2.08 (−2.77, −1.38)
European Union	131 (122, 140)	158 (150, 166)	20.61%	0.08 (0.08, 0.09)	0.12 (0.11, 0.12)	1.82 (1.42, 2.22)

**Table 4:** Disability-adjusted life years of ILD and PS in G20 countries and global average, and temporal trends from 1990 to 2021.

Location	Cases		1990–2021 change (%)	Age-standardized DALYs rate, per 100000 (95% UI)		1990–2021 EAPC (95% CI)
	1990	2021		1990	2021	
China	14,208 (10,186, 21,137)	10,476 (7,190, 14,613)	−26.27%	2.8 (2.0, 4.1)	2.0 (1.4, 2.9)	−1.2 (−1.3, −1.2)
Global	149,440 (113,475, 194,398)	236,946 (190,514, 282,730)	58.56%	7.3 (5.6, 9.5)	8.0 (6.4, 9.5)	0.3 (0.2, 0.4)
Indonesia	1711 (479, 5,108)	3,425 (1,055, 8,783)	100.18%	2.3 (0.7, 6.9)	3.0 (0.9, 7.7)	1.1 (1.0, 1.2)
Russian Federation	4,275 (3,215, 5,276)	1945 (1,618, 2,376)	−54.50%	6.9 (5.2, 8.5)	3.5 (2.9, 4.3)	−3.1 (−3.7, −2.5)
Japan	4,004 (3,326, 4,907)	3,212 (2,756, 3,786)	−19.78%	9.0 (7.5, 11.0)	9.4 (8.2, 11.1)	0.0 (−0.2, 0.3)
Republic of Korea	1,214 (737, 1899)	1,134 (734, 1,645)	−6.59%	6.0 (3.7, 9.4)	6.5 (4.2, 9.5)	0.5 (0.4, 0.6)
Australia	179 (146, 221)	756 (633, 890)	322.35%	2.6 (2.1, 3.2)	8.3 (6.9, 9.7)	3.9 (3.1, 4.8)
France	867 (709, 1,055)	1,409 (1,189, 1,670)	62.51%	3.9 (3.2, 4.7)	7.0 (5.9, 8.2)	2.4 (2.0, 2.8)
Germany	1,284 (1,012,1,586)	1,502 (1,261, 1775)	16.98%	4.2 (3.3, 5.2)	5.6 (4.7, 6.6)	2.0 (1.6, 2.4)
Italy	697 (505, 962)	1,491 (1,339, 1,677)	113.92%	3.3 (2.4, 4.6)	9.1 (8.2, 10.2)	4.0 (3.0, 5.0)
United Kingdom	1,208 (1,003, 1,493)	2,950 (2,768, 3,178)	144.21%	5.9 (4.9, 7.2)	12.9 (12.1, 13.9)	4.0 (3.3, 4.7)
Argentina	1839 (1,524, 2,201)	2,416 (1991, 2,891)	31.38%	15.5 (12.9, 18.5)	13.8 (11.3, 16.5)	0.0 (−0.3, 0.3)
Canada	1,036 (849, 1,259)	1904 (1,606, 2,271)	83.78%	8.9 (7.2, 10.8)	15.2 (12.8, 18.2)	1.9 (1.6, 2.1)
United States of America	19,818 (18,016, 22,168)	17,713 (15,940, 19,773)	−10.62%	18.5 (16.9, 20.7)	15.6 (14.0, 17.4)	−0.5 (−0.6, −0.5)
Mexico	3,626 (3,376, 3,912)	11,006 (9,826, 12,277)	203.53%	11.6 (10.9, 12.6)	21.7 (19.3, 24.2)	2.1 (1.8, 2.4)
Brazil	6,679 (6,104, 7,362)	11,297 (10,550, 12,109)	69.14%	11.4 (10.4, 12.6)	12.9 (12.1, 13.9)	−0.2 (−0.5, 0.2)
Saudi Arabia	1,127 (538, 2039)	5,611 (3,227, 9,191)	397.87%	18.2 (8.8, 32.6)	27.2 (15.5, 44.6)	2.0 (1.7, 2.3)
Turkey	1,438 (722, 2,593)	2,135 (1,289, 3,345)	48.47%	6.5 (3.3, 11.6)	6.6 (4.0, 10.4)	0.6 (0.4, 0.9)
India	33,358 (15,509, 57,578)	65,063 (42,156, 90,945)	95.04%	10.8 (5.0, 18.5)	11.1 (7.2, 15.6)	0.0 (−0.2, 0.3)
South Africa	2088 (1,157, 2,916)	2,219 (1,569, 3,257)	6.27%	15.1 (8.4, 21.0)	8.9 (6.2, 13.0)	−1.9 (−2.6, −1.3)
European Union	9,913 (8,738, 11,468)	11,294 (10,300, 12,528)	13.93%	6.2 (5.5, 7.2)	8.3 (7.5, 9.1)	1.5 (1.2, 1.9)

In 1990, China ranked second in ASPR among G20 countries, following Turkey, and maintained this position in 2021, second only to France (Table 5). The number of incident lung cancer cases in China was 11,847.0 (95% UI: 10,038.1–13,826.7) in 1990, which increased to 12,363.9 (95% UI: 9,857.2–15,097.3) in 2021. The ASIR of lung cancer in China rose from the second highest in 1990 to the highest among G20 nations in 2021 (Table 6). Regarding mortality, there were 10,611.5 lung cancer deaths (95% UI: 9,005.6–12,396.3) in 1990, which decreased to 9,602.7 (95% UI: 7,610.4–11,776.2) in 2021. Over the same period, China’s ASDR for lung cancer increased from the second to the highest position (Table 7). Globally, DALYs due to ILD and PS were approximately 1,335,868.6 thousand (95% UI: 1,229,640.3–1,453,509.1) in 1990 and 1,291,020.4 thousand (95% UI: 1,156,482.7–1,426,189.5) in 2021. In 1990, Turkey had the highest age-standardized DALY rate per 100,000 population at 142.70 (95% UI: 91.30–206.40), followed by China with 122.60 (95% UI: 104.00–143.20). By 2021, China recorded the highest age-standardized DALY rate at 101.30 (95% UI: 80.40–124.30), while Turkey had the second highest rate of 70.90 (95% UI: 48.90–98.60) (Table 8).

**Table 5:** Prevalence of lung cancer in G20 countries and global average, and temporal trends from 1990 to 2021.

Location	Cases		1990–2021 change (%)	Age-standardized prevalence rate, per 100000 (95% UI)		1990–2021 EAPC (95% CI)
	1990	2021		1990	2021	
China	25,458 (21,579, 29,740)	33,250 (26,606, 40,511)	30.61%	5.20 (4.40, 6.10)	6.10 (4.90, 7.40)	0.22 (0.02, 0.41)
Global	60,366 (56,001, 65,149)	71,750 (64,273, 79,637)	18.86%	3.10 (2.90, 3.30)	2.40 (2.20, 2.70)	−1.04 (−1.15, −0.93)
Indonesia	1,440 (1,062, 1801)	2,795 (1923, 3,906)	94.10%	2.10 (1.60, 2.60)	2.40 (1.70, 3.40)	0.46 (0.32, 0.60)
Russian Federation	2,521 (2,428, 2,621)	1,378 (1,270, 1,484)	−45.34%	4.00 (3.80, 4.10)	2.40 (2.20, 2.50)	−1.68 (−1.89, −1.47)
Japan	1,552 (1,453, 1,662)	1,022 (870, 12,010)	−34.15%	3.40 (3.20, 3.70)	2.80 (2.40, 3.40)	0.04 (−0.65, 0.73)
Republic of Korea	726 (5,510, 913)	616 (444, 855)	−15.15%	3.70 (2.80, 4.60)	3.40 (2.40, 4.70)	−0.27 (−0.41, −0.13)
Australia	189 (159, 225)	266 (208, 336)	40.74%	2.70 (2.30, 3.30)	2.70 (2.20, 3.50)	0.60 (−0.05, 1.24)
France	1,084 (896, 1,306)	1,472 (1,169, 1825)	35.79%	4.80 (3.90, 5.70)	7.00 (5.60, 8.70)	1.92 (1.67, 2.17)
Germany	1,164 (945, 1,391)	762 (600, 950)	−34.54%	3.80 (3.10, 4.50)	2.60 (2.10, 3.30)	−0.52 (−0.94, −0.11)
Italy	672 (618, 729)	396 (351, 447)	−41.07%	3.30 (3.00, 3.60)	2.30 (2.00, 2.60)	−1.00 (−1.21, −0.79)
United Kingdom	500 (485, 517)	528 (503, 553)	5.6%	2.50 (2.40, 2.50)	2.20 (2.10, 2.30)	0.42 (0.07, 0.76)
Argentina	437 (366, 524)	337 (276, 410)	−22.88%	3.80 (3.20, 4.60)	1.90 (1.60, 2.30)	−2.23 (−2.46, −1.99)
Canada	510 (444, 585)	335 (274, 406)	−34.31%	4.30 (3.70, 4.90)	2.60 (2.10, 3.10)	−2.36 (−2.65, −2.07)
United States of America	4,727 (4,564, 4,895)	2,485 (2,364, 2,611)	−47.43%	4.40 (4.20, 4.50)	2.20 (2.10, 2.30)	−2.52 (−2.73, −2.30)
Mexico	526 (504, 547)	593 (532, 658)	12.74%	1.80 (1.70, 1.80)	1.20 (1.00, 1.30)	−1.23 (−1.43, −1.04)
Brazil	1,011 (946, 1,082)	1,396 (1,297, 1,507)	38.08%	1.90 (1.70, 2.00)	1.60 (1.50, 1.70)	−0.61 (−0.73, −0.49)
Saudi Arabia	27 (17, 42)	131 (77, 215)	385.19%	0.50 (0.30, 0.70)	0.60 (0.30, 1.00)	1.12 (0.73, 1.51)
Turkey	1,177 (755, 1727)	983 (683, 1,361)	−16.48%	5.70 (3.60, 8.40)	3.00 (2.10, 4.20)	−2.25 (−2.54, −1.97)
India	2,548 (2,154, 3,013)	5,640 (4,787, 6,638)	121.35%	0.80 (0.70, 1.00)	1.00 (0.80, 1.20)	0.73 (0.48, 0.98)
South Africa	416 (343, 495)	412 (342, 508)	−0.96%	3.30 (2.70, 3.90)	1.60 (1.30, 2.00)	−2.59 (−3.13, −2.04)
European Union	6,668 (6,290, 7,053)	4,628 (4,176, 5,131)	−30.59%	4.20 (3.90, 4.40)	3.20 (2.80, 3.50)	−0.71 (−0.84, −0.58)

**Table 6:** Incidence of lung cancer in G20 countries and global average, and temporal trends from 1990 to 2021.

Location	Cases		1990–2021 change (%)	Age-standardized incidence rate, per 100000 (95% UI)		1990–2021 EAPC (95% CI)
	1990	2021		1990	2021	
China	11,847 (10,038, 13,827)	12,364 (9,857, 15,097)	4.36%	2.40 (2.00, 2.80)	2.30 (1.80, 2.80)	−0.52 (−0.71, −0.34)
Global	26,791 (24,734, 29,038)	28,004 (25,084, 31,022)	4.53%	1.40 (1.30, 1.50)	0.90 (0.80, 1.00)	−1.70 (−1.83, −1.56)
Indonesia	676 (500, 845)	1,286 (885, 1796)	90.24%	1.00 (0.70, 1.30)	1.10 (0.80, 1.60)	0.36 (0.20, 0.52)
Russian Federation	1,086 (1,048, 1,126)	513 (474, 553)	−52.76%	1.70 (1.60, 1.80)	0.90 (0.80, 0.90)	−2.22 (−2.42, −2.01)
Japan	558 (532, 587)	259 (231, 297)	−53.58%	1.20 (1.20, 1.30)	0.70 (0.60, 0.80)	−1.27 (−1.71, −0.84)
Republic of Korea	318 (245, 401)	169 (123, 232)	−46.86%	1.70 (1.20, 2.10)	0.90 (0.70, 1.30)	−2.17 (−2.32, −2.01)
Australia	63 (53, 74)	68 (54, 85)	7.94%	0.90 (0.80, 1.10)	0.70 (0.60, 0.90)	−0.35 (−0.72, 0.02)
France	425 (356, 510)	354 (286, 436)	−16.71%	1.80 (1.50, 2.20)	1.70 (1.40, 2.10)	−0.02 (−0.15, 0.11)
Germany	458 (374, 543)	202 (159, 249)	−55.90%	1.50 (1.20, 1.80)	0.70 (0.50, 0.90)	−2.10 (−2.34, −1.86)
Italy	283 (263, 306)	132 (118, 146)	−53.36%	1.40 (1.30, 1.50)	0.80 (0.70, 0.80)	−1.93 (−2.13, −1.74)
United Kingdom	204 (198, 210)	173 (166, 180)	−15.20%	1.00 (1.00, 1.00)	0.70 (0.70, 0.80)	−0.39 (−0.73, −0.06)
Argentina	205 (171, 245)	146 (119, 177)	−28.78%	1.80 (1.50, 2.10)	0.80 (0.70, 1.00)	−2.43 (−2.65, −2.20)
Canada	182 (160, 206)	95 (79, 114)	−47.80%	1.50 (1.30, 1.70)	0.70 (0.60, 0.90)	−3.18 (−3.52, −2.84)
United States of America	1,678 (1,623, 1734)	748 (715, 784)	−55.42%	1.50 (1.50, 1.60)	0.60 (0.60, 0.70)	−2.98 (−3.19, −2.77)
Mexico	240 (230, 250)	252 (226, 279)	5%	0.80 (0.80, 0.80)	0.50 (0.40, 0.50)	−1.66 (−1.91, −1.41)
Brazil	471 (440, 503)	622 (577, 672)	32.06%	0.90 (0.80, 0.90)	0.70 (0.60, 0.70)	−0.82 (−1.04, −0.61)
Saudi Arabia	13 (8, 20)	60 (35, 97)	361.54%	0.20 (0.20, 0.30)	0.30 (0.20, 0.40)	0.45 (0.10, 0.79)
Turkey	555 (356, 815)	442 (306, 610)	−20.36%	2.70 (1.80, 4.00)	1.30 (1.00, 1.90)	−2.51 (−2.78, −2.24)
India	1,198 (1,012, 1,417)	2,585 (2,193, 3,049)	115.78%	0.40 (0.30, 0.50)	0.40 (0.40, 0.50)	0.43 (0.05, 0.81)
South Africa	198 (163, 235)	196 (162, 241)	−1.01%	1.60 (1.30, 1.90)	0.80 (0.60, 1.00)	−2.50 (−3.08, −1.92)
European Union	2,764 (2,622, 2,912)	1,386 (1,275, 1,513)	−49.86%	1.70 (1.60, 1.80)	0.90 (0.90, 1.00)	−1.97 (−2.11, −1.83)

**Table 7:** Mortality of lung cancer in G20 countries and global average, and temporal trends from 1990 to 2021.

Location	Cases		1990–2021 change (%)	Age-standardized Deaths rate, per 100000 (95% UI)		1990–2021 EAPC (95% CI)
	1990	2021		1990	2021	
China	10,612 (9,006, 12,396)	9,603 (7,610, 11,776)	−9.51%	2.10 (1.80, 2.50)	1.80 (1.40, 2.20)	−0.98 (−1.18, −0.78)
Global	23,309 (21,480, 25,336)	22,622 (20,241, 24,998)	−2.95%	1.20 (1.10, 1.30)	0.70 (0.70, 0.80)	−1.87 (−2.02, −1.72)
Indonesia	624 (459, 780)	1,159 (795, 1,613)	85.74%	0.90 (0.70, 1.20)	1.00 (0.70, 1.40)	0.43 (0.22, 0.63)
Russian Federation	936 (901, 971)	401 (371, 432)	−57.16%	1.50 (1.40, 1.50)	0.70 (0.70, 0.70)	−2.52 (−2.76, −2.29)
Japan	387 (374, 402)	132 (127, 137)	−65.89%	0.80 (0.80, 0.90)	0.30 (0.30, 0.40)	−2.84 (−3.17, −2.51)
Republic of Korea	272 (210, 344)	92 (69, 122)	−66.18%	1.40 (1.10, 1.80)	0.50 (0.40, 0.70)	−3.66 (−3.85, −3.47)
Australia	41 (35, 48)	35 (29, 43)	−14.63%	0.60 (0.50, 0.70)	0.30 (0.30, 0.40)	−1.66 (−1.97, −1.34)
France	326 (273, 391)	179 (148, 214)	−45.09%	1.40 (1.20, 1.70)	0.90 (0.70, 1.00)	−1.64 (−1.80, −1.48)
Germany	358 (294, 425)	125 (101, 154)	−65.08%	1.20 (1.00, 1.40)	0.40 (0.30, 0.50)	−2.87 (−3.14, −2.60)
Italy	227 (212, 243)	89 (82, 96)	−60.80%	1.10 (1.00, 1.20)	0.50 (0.50, 0.60)	−2.31 (−2.51, −2.12)
United Kingdom	161 (157, 166)	117 (113, 121)	−27.33%	0.80 (0.80, 0.80)	0.50 (0.50, 0.50)	−1.04 (−1.39, −0.69)
Argentina	180 (150, 215)	122 (100, 148)	−32.22%	1.60 (1.30, 1.90)	0.70 (0.60, 0.80)	−2.65 (−2.87, −2.42)
Canada	135 (119, 152)	59 (51, 69)	−56.30%	1.10 (1.00, 1.30)	0.40 (0.40, 0.50)	−3.86 (−4.22, −3.50)
United States of America	1,253 (1,214, 1,293)	506 (484, 529)	−59.62%	1.20 (1.10, 1.20)	0.40 (0.40, 0.50)	−3.35 (−3.56, −3.14)
Mexico	220 (211, 229)	219 (198, 242)	−0.45%	0.70 (0.70, 0.80)	0.40 (0.40, 0.50)	−2.03 (−2.32, −1.74)
Brazil	425 (397, 455)	537 (499, 579)	26.35%	0.80 (0.70, 0.80)	0.60 (0.50, 0.70)	−0.91 (−1.13, −0.69)
Saudi Arabia	12 (7, 18)	53 (31, 88)	341.67%	0.20 (0.10, 0.30)	0.20 (0.10, 0.40)	1.07 (0.39, 1.75)
Turkey	513 (328, 743)	398 (274, 553)	−22.42%	2.50 (1.60, 3.60)	1.20 (0.90, 1.70)	−2.53 (−2.81, −2.25)
India	1,099 (928, 1,302)	2,306 (1954, 2,728)	109.83%	0.40 (0.30, 0.40)	0.40 (0.40, 0.50)	0.00 (0.00, 0.00)
South Africa	177 (146, 211)	172 (143, 212)	−2.82%	1.40 (1.10, 1.70)	0.70 (0.60, 0.90)	−2.68 (−3.30, −2.07)
European Union	2,225 (2,113, 2,343)	893 (826, 960)	−59.87%	1.40 (1.30, 1.40)	0.60 (0.60, 0.70)	−2.67 (−2.84, −2.51)

**Table 8:** Disability-adjusted life years of lung cancer in G20 countries and global average, and temporal trends from 1990 to 2021.

Location	Cases		1990–2021 change (%)	Age-standardized DALYs rate, per 100000 (95% UI)		1990–2021 EAPC (95% CI)
	1990	2021		1990	2021	
China	609,492 (516,860, 712,709)	542,424 (430,333, 664,815)	−11.00%	122.60 (104.00, 143.20)	101.30 (80.40, 124.30)	−1.01 (−1.19, −0.83)
Global	1,335,869 (1,229,640, 1,453,509)	1,291,020 (1,156,483, 1,426,190)	−3.36%	67.40 (62.10, 73.20)	43.40 (38.90, 47.90)	−1.70 (−1.81, −1.58)
Indonesia	36,684 (26,874, 45,898)	67,347 (46,199, 93,781)	83.59%	52.90 (38.90, 66.00)	59.10 (40.50, 82.20)	0.31 (0.18, 0.45)
Russian Federation	52,412 (50,529, 54,396)	22,307 (20,613,24,072)	−57.44%	82.90 (79.90, 86.00)	38.40 (35.50, 41.50)	−2.53 (−2.77, −2.29)
Japan	21,589 (20,844, 22,389)	7,465 (7,180, 7,755)	−65.42%	47.40 (45.70,49.10)	20.80 (20.00,21.70)	−2.64 (−2.85, −2.42)
Republic of Korea	15,766 (12,145, 19,917)	5,162 (3,856, 6,911)	−67.26%	80.30 (62.00, 101.50)	28.70 (21.30, 38.50)	−3.72 (−3.93, −3.52)
Australia	2,332 (1980, 2,734)	1973 (1,627, 2,390)	−15.39%	34.10 (28.90, 40.00)	20.60 (17.00, 25.00)	−1.51 (−1.73, −1.29)
France	18,027 (15,135, 21,628)	9,993 (8,231, 11,978)	−44.57%	79.20 (66.50, 94.90)	47.80 (39.40, 57.40)	−1.67 (−1.78, −1.57)
Germany	19,909 (16,387, 23,686)	7,035 (5,641, 8,650)	−64.66%	65.40 (53.90, 77.90)	24.80 (19.90, 30.50)	−2.83 (−3.03, −2.62)
Italy	12,836 (12,006, 13,747)	4,998 (4,603, 5,416)	−61.06%	62.60 (58.60, 67.10)	28.80 (26.50, 31.30)	−2.46 (−2.60, −2.32)
United Kingdom	9,007 (8,781, 9,269)	6,519 (6,300, 6,751)	−27.62%	44.30 (43.10, 45.50)	27.70 (26.80, 28.70)	−0.91 (−1.20, −0.61)
Argentina	10,116 (8,450, 12,121)	7,018 (5,715, 8,518)	−30.62%	87.70 (73.30, 105.10)	40.00 (32.60, 48.60)	−2.48 (−2.71, −2.26)
Canada	7,557 (6,666, 8,518)	3,356 (2,866, 3,912)	−55.59%	63.60 (56.10, 71.80)	26.00 (22.20, 30.40)	−3.72 (−4.08, −3.36)
United States of America	69,801 (67,634, 72,034)	28,688 (27,451, 29,998)	−58.90%	64.80 (62.70, 66.80)	25.00 (23.90, 26.10)	−3.28 (−3.42, −3.15)
Mexico	13,109 (12,592, 13,635)	12,854 (11,588, 14,168)	−1.95%	42.60 (40.90, 44.30)	25.40 (22.90, 28.10)	−1.73 (−1.91, −1.55)
Brazil	24,610 (23,013, 26,352)	30,900 (28,710, 33,343)	25.56%	44.00 (41.20, 47.10)	34.80 (32.30, 37.50)	−0.77 (−0.86, −0.68)
Saudi Arabia	665 (406, 1,033)	2,978 (1755, 4,934)	347.82%	11.80 (7.20, 18.20)	13.40 (7.90, 22.30)	0.79 (0.49, 1.10)
Turkey	30,101 (19,291, 43,576)	23,078 (15,899, 32,072)	−23.33%	142.70 (91.30, 206.40)	70.90 (48.90, 98.60)	−2.48 (−2.76, −2.20)
India	64,131 (54,081, 76,061)	133,254 (112,789, 158,191)	107.78%	20.90 (17.60, 24.70)	22.80 (19.30, 27.10)	0.35 (0.17, 0.52)
South Africa	9,942 (8,227, 11,851)	9,624 (7,995, 11,881)	−3.20%	77.90 (64.30, 92.90)	38.10 (31.70, 47.10)	−2.67 (−3.25, −2.08)
European Union	124,161 (117,931, 130,735)	50,053 (46,298, 53,859)	−59.69%	77.30 (73.40, 81.40)	34.40 (31.80, 37.10)	−2.66 (−2.75, −2.56)

### 4.2 Temporal trends in ILD and PS lung cancer

Among G20 nations, Saudi Arabia exhibited the most pronounced increase in ILD and PS ASPR (EAPC = 1.6; 95% CI: 1.5, 1.7), followed by Germany and Turkey (both EAPC = 1.0; 95% CI: 0.4, 1.7; Turkey 95% CI: 0.7, 1.3). China showed a declining ASPR trend (EAPC = −2.5; 95% CI: −2.9, −2.1), representing the second largest decrease after Brazil (EAPC = −2.6; 95% CI: −3.2, −2.1) (Table 1). For ASIR, Saudi Arabia demonstrated the highest increase (EAPC = 1.7; 95% CI: 1.6, 1.8), with Turkey ranking second (EAPC = 1.1; 95% CI: 0.8, 1.4). Conversely, China’s ASIR declined (EAPC = −2.1; 95% CI: −2.5, −1.8), marking the second largest reduction behind Brazil (EAPC = −2.6; 95% CI: −3.2, −2.0) (Table 2). Between 1990 and 2021, China, Russia, Argentina, the US, Brazil, and South Africa experienced declining ASDR for ILD and PS. Conversely, Indonesia, Australia, the EU, France, Italy, Japan, Turkey, the UK, Canada, Germany, South Korea, Mexico, and Saudi Arabia showed increasing trends. Italy had the steepest ASDR increase (EAPC = 6.33; 95% CI: 4.87, 7.82), while Russia showed the sharpest decline (EAPC = −3.91; 95% CI: −4.77, −3.04). China ranked fourth in ASDR reduction (EAPC = −0.55; 95% CI: −0.71, −0.39) (Table 3). China’s age-standardized DALY rate for ILD and PS decreased (EAPC = −1.2; 95% CI: −1.3, −1.2), representing the third largest reduction among G20 nations after Russia and South Africa (Table 4).

France showed the most substantial increase in lung cancer ASPR (EAPC = 1.92; 95% CI: 1.67, 2.17), followed by Saudi Arabia (EAPC = 1.12; 95% CI: 0.73, 1.51). China ranked 7th (EAPC = 0.22; 95% CI: 0.02, 0.41), while South Africa exhibited the steepest decline (EAPC = −2.59; 95% CI: −3.13, −2.04) (Table 5). Saudi Arabia had the highest ASIR increase (EAPC = 0.45; 95% CI: 0.10, 0.79), with India ranking second (EAPC = 0.43; 95% CI: 0.05, 0.81). China demonstrated a declining ASIR trend (EAPC = −0.52; 95% CI: −0.71, −0.34), ranking 7th in magnitude of change. Canada showed the most significant reduction (EAPC = −3.18; 95% CI: −3.52, −2.84) (Table 6). For lung cancer ASDR (1990–2021), only Indonesia and Saudi Arabia showed increases, India remained stable, while all other G20 nations declined. Canada had the sharpest ASDR reduction (EAPC = −3.86; 95% CI: −4.22, −3.50), followed by South Korea (EAPC = −3.66; 95% CI: −3.85, −3.47) (Table 7). China’s age-standardized DALY rate for lung cancer decreased (EAPC = −1.01; 95% CI: −1.19, −0.83), ranking 6th among G20 nations (Table 8).

### 4.3 Decomposition analysis of changes in ILD and PS lung Cancer incidence, prevalence, and DALYs

**Figure 1:**
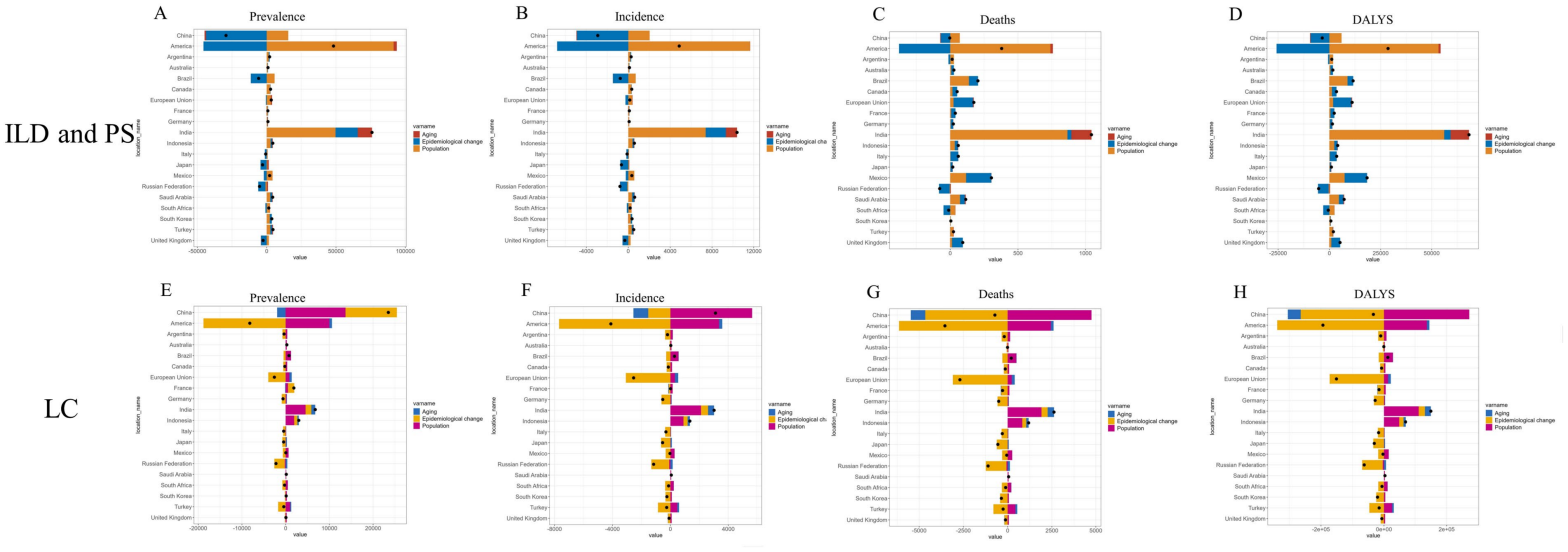
Decomposition analysis of changes in ILD and PS cancer metrics from 1990 to 2021. The black dots represent the overall change values attributable to population growth, aging, and epidemiological transitions. Positive values indicate favorable contributions, while negative values correspond to adverse effects. Panels **A** and **E** depict prevalence; **B** and **F** depict incidence; **C** and **G** depict mortality; **D** and **H** depict DALYs. ILD and PS, interstitial lung disease; LC, lung cancer.

In China, population growth contributed to a 52.86% increase in ILD and PS prevalent cases, while epidemiological transitions offset this by reducing cases by 149.08%, and population aging further reduced cases by 3.79%. In the United States, population growth increased prevalent cases by 189.97%, partially offset by epidemiological transitions (−94.61%), with population aging contributing a 4.64% increase. In India, population growth (+65.32%), epidemiological transitions (+21.14%), and population aging (+13.54%) all drove increases in prevalent cases (Figure 1A; Supplementary Table S1). Population growth drove a 70.02% increase in incident ILD and PS cases in China, counteracted by epidemiological transitions (−168.13%) and a minor reduction from population aging (−1.89%). The US saw population growth contribute a 239.36% increase, largely offset by epidemiological transitions (−139.63%), with minimal impact from population aging (+0.27%). In India, incident cases increased due to population growth (+71.13%), epidemiological transitions (+18.66%), and population aging (+10.21%) (Figure 1B; Supplementary Table S2). The rise in absolute ILD and PS deaths and DALYs in China was primarily driven by population growth, whereas epidemiological transitions and population aging were the main factors limiting this increase (Figures 1C,D; Supplementary Table S3, S4).

In China, population growth (+58.31%) and epidemiological transitions (+49.96%) increased prevalent lung cancer cases, partially offset by population aging (−8.28%). In the US, population growth increased cases (+121.83%), counteracted by a larger reduction from epidemiological transitions (−229.05%), while population aging contributed a 7.22% increase. In the European Union, population growth (+33.65%) and aging (+20.59%) increased cases, significantly offset by epidemiological transitions (−154.24%) (Figure 1E; Supplementary Table S5). China experienced a substantial increase in incident lung cancer cases due to population growth (+181.95%), partially counterbalanced by epidemiological transitions (−49.10%) and population aging (−32.85%). In the US, population growth contributed an 81.79% increase, outweighed by epidemiological transitions (−187.03%), with a minor increase from population aging (+5.23%). In the EU, population growth (+12.27%) and aging (+9.00%) increased incidence, significantly offset by epidemiological transitions (−121.28%) (Figure 1F; Supplementary Table S6). The increase in absolute lung cancer deaths and DALYs in China was predominantly attributable to population growth, while epidemiological transitions and population aging acted as the principal mitigating factors (Figures 1G,H; Supplementary Tables S7, S8).

### 4.4 Projection of ILD and PS lung cancer in China

**Figure 2:**
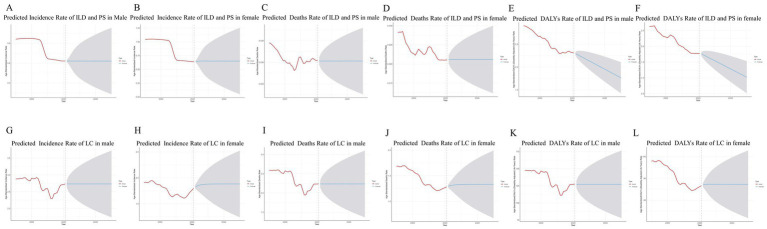
The time trends of ILD and PS lung cancer in China from 1990 to 2021, stratified by gender. **(A)** Time trend of incidence rate of ILD and PS prediction for males in 2050. **(B)** Time trend of incidence rate of ILD and PS prediction for females in 2050. **(C)** Time trend of deaths rate of ILD and PS prediction for males in 2050. **(D)** Time trend of deaths rate of ILD and PS prediction for females in 2050. **(E)** Time trend of DALY rate of ILD and PS prediction for males by 2050. **(F)** Time trend of DALY rate of ILD and PS prediction for females in 2050. **(G)** Time trend of incidence rate of lung cancer and prediction for males in 2050. **(H)** Time trend of incidence rate of lung cancer and prediction for females in 2050. **(I)** Time trend of deaths rate of lung cancer and prediction for males in 2050. **(J)** Time trend of deaths rate of lung cancer and prediction for females in 2050. **(K)** Time trend of DALY rate of lung cancer and prediction for males by 2050. **(L)** Time trend of DALY rate of lung cancer and prediction for females in 2050.

Using autoregressive integrated moving average (ARIMA) models, we fitted ASIR, ASDR, and DALY rates for ILD and PS stratified by sex from 1990 to 2021 and projected values to 2050. By 2050, the ASIR is projected to reach 0.631 (95% UI: 0.553–0.710) per 100,000 males (Figure 2A) and 0.651 (95% UI: 0.549–0.749) per 100,000 females (Figure 2B; Supplementary Table S9). The ASDR is forecasted at 0.030 (95% UI: 0.028–0.033) per 100,000 males (Figure 2C) and 0.021 (95% UI: 0.017–0.024) per 100,000 females (Figure 2D; Supplementary Table S10). Age-standardized DALY rates are projected to be 1.766 (95% UI: 1.157–2.357) per 100,000 males (Figure 2E) and 1.021 (95% UI: 0.561–1.857) per 100,000 females (Figure 2F; Supplementary Table S11).

By 2050, the ASIR is projected to reach 2.730 (95% UI: 2.445–3.015) per 100,000 males (Figure 2G) and 1.882 (95% UI: 1.741–1.964) per 100,000 females (Figure 2H; Supplementary Table S12). The ASDR is forecasted at 2.235 (95% UI: 1.986–2.484) per 100,000 males (Figure 2I) and 1.333 (95% UI: 1.211–1.413) per 100,000 females (Figure 2J; Supplementary Table S13). Age-standardized DALY rates are projected to be 127.212 (95% UI: 113.029–141.395) per 100,000 males (Figure 2K) and 74.581 (95% UI: 68.024–81.080) per 100,000 females (Figure 2L; Supplementary Table S14).

## 5 Discussion

ILD and PS lung cancer represent significant global health challenges, with particularly pronounced disease burdens in China. Moreover, ILD and PS continues to impose substantial health losses despite recent epidemiological transitions (23). In our country, lung cancer has emerged as the primary cause of death from cancer (24). Systematically evaluating the long-term epidemiological trajectories of these diseases in China and identifying critical control points through cross-national comparisons hold pressing practical significance for developing targeted prevention strategies. Leveraging GBD 2021 data, this study presents the first comprehensive assessment of ILD and PS lung cancer burden metrics—including incidence, prevalence, deaths and DALYs-among adolescents and young adults (aged 15–39 years) across G20 member states from 1990 to 2021. We further quantified contributing factors and developed projection models. A comparative analysis was conducted benchmarking G20 nations’ disease indicators against other members. The G20, serving as the premier forum for international economic cooperation between major advanced and emerging economies, exhibits disease burden profiles intrinsically linked to members’ distinct economic development levels and population health statuses (25). Analysis indicates that the majority of G20 member states confront significant challenges stemming from non-communicable diseases (NCDs), exemplified by ILD and PS lung cancer. Given the widespread prevalence of accelerating population aging and the mounting burden of NCDs across these nations, the G20 is positioned to assume a pivotal role in advancing global health governance and enhancing global health outcomes.

In China, the incidence, prevalence, deaths, and DALYs associated with ILD and PS among adolescents and young adults exhibited a gradual decline from 1990 to 2021, attributable to the synergistic contributions of multiple factors. Foremost among these driving factors were substantial advancements in the early diagnosis of ILD and PS, particularly the widespread clinical implementation of high-resolution computed tomography (HRCT) (26) and improved accuracy in serum biomarker detection (23), which substantially reduced rates of misdiagnosis and underdiagnosis. Furthermore, optimizations in treatment paradigms yielded substantial impact: the evidence-guided application of antifibrotic agents (e.g., pirfenidone, nintedanib), the adoption of personalized immunosuppressive regimens, and refinements in lung transplantation technique collectively contributed to improved disease outcomes (27). Enhanced preventive measures proved equally pivotal, encompassing the implementation of public health tobacco control policies that mitigated environmental exposure risks and the refinement of occupational dust exposure standards that reduced contact with pathogenic agents (28). The 2003 SARS outbreak served as a catalyst for reforming global surveillance systems for respiratory infectious diseases. In its aftermath, the World Health Organization (WHO) enhanced symptom-based screening procedures and international notification protocols (29). These improvements have indirectly facilitated earlier identification and management of interstitial lung disease (ILD and PS) in adolescent and young adult populations.

Furthermore, the establishment of respiratory disease registries globally facilitated screening of high-risk populations (30), while enhanced public health awareness accelerated health-seeking behaviors. Consequently, the integration of advances in medical technology, optimized clinical management strategies, and public health interventions collectively drove favorable trends in ILD and PS related health metrics for this demographic.

Within China, ASPR, ASIR, ASDR and DALYs related to lung cancer among adolescents and young adults considerably exceeded corresponding rates in G20 nations, a notably concerning trend. This disparity may be attributable to factors including tobacco use, ambient air pollution, and genetic susceptibility. Tobacco exposure constituted the primary driver of escalating lung cancer morbidity and mortality risks. Epidemiological studies confirm that smoking prevalence remains persistently high among Chinese males (31), with a significant upward trend observed among females (32). Notably, the rising prevalence of tobacco use among adolescents warrants heightened concern, with vocational high school students exhibiting a smoking rate of 21.2% in 2021 (Chinese Center for Disease Control and Prevention data). Concurrently, more than one-third of non-smokers and youngsters experienced detrimental exposure to secondhand smoke (33). Despite nationwide implementation of tobacco control policies, their efficacy has fallen short of intended targets. Approximately 90% of smokers lack access to evidence-based cessation interventions, resulting in persistently low rates of sustained abstinence. Moreover, merely 16.1% of current smokers report cessation intentions (34). Furthermore, significant operational deficiencies persist within primary care smoking cessation services. Both awareness and utilization rates of cessation clinics and quitlines remain suboptimal, while the proportion of clinicians providing evidence-based cessation guidance accounts for only half of that observed in developed nations (35). In contrast, G20 nations have achieved significant progress in tobacco control through legislative reinforcement. Countries including the United States, Japan, and Brazil demonstrate sustained declines in per capita tobacco consumption. Notably, Australia implemented comprehensive measures—encompassing nationwide smoke-free legislation, elevated tobacco excise taxes, mandatory graphic health warnings, and comprehensive bans on tobacco advertising and e-cigarettes—which reduced its smoking prevalence from 43.4% in 2001 to 12.8% in 2017 (36). These outcomes underscore the imperative for the Chinese government to establish a scientifically grounded tobacco control framework prioritizing increased tobacco taxation and standardized graphic health warnings on packaging, thereby mitigating tobacco-attributable health burdens.

Air pollution exposure and genetic susceptibility represent key etiological determinants of lung cancer. China’s rapid industrialization and urbanization have driven substantial increases in industrial energy consumption and pollutant emissions, resulting in consistently elevated levels of ambient fine particulate matter (PM₂.₅) pollution—ranking among the highest within G20 nations (37). Following the implementation of the Air Pollution Prevention and Control Action Plan in 2013, the national annual average PM₂.₅ concentration decreased from 67.4 μg/m^3^ to 45.5 μg/m^3^ by 2017 (38). Nevertheless, these levels remained 2–4 times higher than contemporary levels in Australia (12.1–21.7 μg/m^3^). Furthermore, 70.7% of cities (239 out of 338) still faced severe air pollution that year, according to data from China’s Ministry of Ecology and Environment (39). While some G20 nations have achieved significant pollution reduction through vehicle emission controls and clean energy policies, China urgently requires strengthened industrial energy consumption constraints and accelerated transformation of its energy mix. Moreover, household fuel pollution poses a substantial threat. A coal-dominated residential energy structure results in China accounting for 3% (40) of the global population exposed to household air pollution. Promoting clean fuels (e.g., liquefied petroleum gas or natural gas), coupled with improved ventilation systems and optimized cooking practices, could significantly reduce exposure to indoor air pollutants. Genetically, the Chinese population exhibits heightened genetic susceptibility to lung cancer. This is evidenced by a significantly higher prevalence of pathogenic EGFR gene mutations in Chinese patients with non-small cell lung cancer (NSCLC) compared to other ethnic groups (41), underscoring the necessity for precision-based prevention and control strategies.

Additionally, the proportionality contributions of population expansion, aging, and epidemiological transitions to changes in disease burden were measured using decomposition analysis. Beneficial epidemiological changes were the main mediators of the observed decreases in ILD and PS prevalence, incidence, deaths, and DALYs in China. This implies that programs for health promotion and illness prevention can successfully offset the effects of demographic forces (aging and population expansion) on the burden of ILD and PS.

Conversely, persistent increases in lung cancer incidence and prevalence were predominantly driven by population expansion. Empirical studies indicate demographic growth trajectories in numerous nations through 2050, suggesting population-scale effects will continue elevating lung cancer burden for decades. Consequently, integrating population growth as a core determinant is imperative when formulating national health strategies.

Based on ARIMA model projections, ASPR, ASIR, ASDR, and DALY rates for both ILD and PS lung cancer in China are expected to decline gradually over the next 29 years. However, considerable uncertainty exists as evidenced by wide 95% confidence intervals, warranting cautious interpretation of these forecasts.

This study has several limitations inherent to the GBD database. First, as GBD analyses operate at national and regional levels, they preclude assessment of ethnic and racial determinants. Second, heterogeneity in diagnostic criteria across countries may affect data comparability. Moreover, the absence of histology-specific burden and trend data for ILD and PS lung cancer prevented evaluation of subtype-specific epidemiology. GBD estimates primarily synthesize published studies and national reports rather than direct surveillance systems. This methodological approach may raise concerns regarding data completeness, timeliness, and quality. Although GBD modeling employs smoothing techniques and uncertainty intervals to enhance robustness, residual biases from systematic underdiagnosis and misclassification persist. Consequently, cross-national comparability of disease burden estimates remains constrained.

## 6 Conclusion

The disease burden and temporal trends of lung cancer and ILD and PS in China and other G20 countries were thoroughly evaluated in this study between 1990 and 2021. During this period, China demonstrated progressive declines in ASIR, ASPR, ASDR and DALY rates for both conditions, though substantial heterogeneity was observed across G20 countries. Model projections indicate continued gradual reductions in ILD and PS lung cancer burden in China over the next three decades. Given the persistent epidemiological burden, coordinated multinational efforts are imperative to implement evidence-based interventions optimizing early detection pathways and establish collaborative prevention strategies to mitigate disease impact.

## Data Availability

The datasets presented in this study can be found in online repositories. The names of the repository/repositories and accession number(s) can be found in the article/Supplementary material.
